# Dr. Aaron Nelson Braman

**Published:** 1901-08

**Authors:** 


### OBITUARY NOTES.

Dr. Aaron Nelson Braman, U. of B., '51, died at his home in Rochester, N.Y., July 6, 1901.

